# Case report: *Candida blankii* osteo-articular infection in a patient with Chronic Granulomatous Disease

**DOI:** 10.1016/j.mmcr.2024.100682

**Published:** 2024-10-31

**Authors:** Estelle Sabourin, Clémentine De La Porte des Vaux, Nathanaël Veluppillai, Marie-Elisabeth Bougnoux, Eric Dannaoui, Olivier Lortholary

**Affiliations:** a Université Paris Cité, Faculté de Médecine, APHP, Hôpital Necker Enfants-Malades, Hôpital Européen Georges Pompidou, Unité de Parasitologie-Mycologie, Service de Microbiologie, 149 rue de Sèvres, 75015, Paris, France; b Université Paris Cité, Faculté de Médecine, APHP, Hôpital Necker Enfants malades, Service de Maladies Infectieuses et Tropicales, 149 rue de Sèvres, 75015, Paris, France; c Unité Biologie et Pathogénicité fongiques, Département de Mycologie, Institut Pasteur, Paris, France; d Institut Pasteur, Centre National de Référence Mycoses Invasives et Antifongiques, 75015, Paris, France

**Keywords:** *Candida blankii*, Chronic Granulomatous Disease, Osteoarticular infection, Emerging pathogen

## Abstract

*Candida blankii* is a recently reported yeast causing rare cases of fungemia. This species presents high minimum inhibitory concentrations (MICs) to fluconazole and echinocandins. We report an atypical *C. blankii* metacarpophalangeal osteo-articular infection in a patient with Chronic Granulomatous Disease. The patient was initially treated by a combination of voriconazole and 5-fluorocytosine (5-FC). However, the treatment was subsequently changed to posaconazole and then to a combination of caspofungin and 5-FC.

2012 Elsevier Ltd. All rights reserved.

## 1 Introduction

As a result of advances in fungal identification, human infections with *C. blankii* began to be reported in the 2000s. These included invasive fungal infections in mostly immunocompromised patients [[1], [2], [3], [4]] and, in one case, bronchial exacerbations in a cystic fibrosis patient [5]. This yeast presents high minimum inhibitory concentrations (MICs) to fluconazole and echinocandins, complicating the management of these infections. It has been described mainly in South Asia and South America, which raises the question of the emergence of this pathogen in Europe.

We report the case of a metacarpophalangeal osteoarticular infection with *C. blankii* in a patient with Chronic Granulomatous Disease (CGD). This case will address several questions about these rare infections.

## 2 Case presentation

Mrs X is a 70-year-old patient with autosomal recessive CGD (p47-PHOX mutation on chromosome 7), diagnosed in 1968. She is of Greek origin, and lives there for a few months of the year. She was on long-term isavuconazole for secondary prophylaxis following two episodes of pulmonary aspergillosis infections (*Aspergillus fumigatus* in 2003 and *Aspergillus terreus* in 2008).

In November 2022, the patient consulted at the rheumatology clinic with spontaneous pain in the second metacarpal (MCP 2) of the left hand, evolving over a month, without fever or sore. On the hand X-ray, calcifications were observed opposite the medial aspect of the MCP 2, without erosion. At the consultation on D0, isolated painful synovitis of MCP 2 left was clinically observed. There was no history of skin inoculation and no adenopathy. Synovitis was subsequently confirmed by ultrasound, along with the presence of calcification in the joint, without erosion. The patient was initially treated with NSAIDs *per os* for 3 days, followed by corticosteroids *per os* and icing without effect.

A steroid infiltration was then performed at D90, relieving the pain for a few days, before the pain reappeared with a trail of lymphangitis and left epitrochlear adenitis. Following this, a diagnostic synovial puncture was performed on D135 to search for mycobacteria and 16S PCR, both of which were negative. The patient was treated with an injection of cefazolin IV followed by oral cefalexin for 7 days. The course remained unfavorable, with the onset of pain, oedema and erythema.

**Fig. 1:**
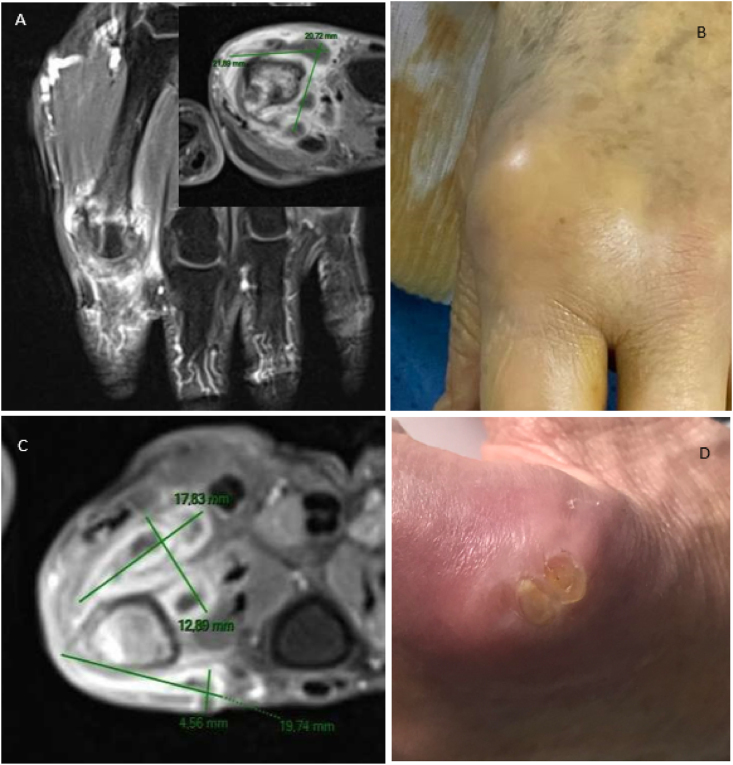
MRI and clinical evolution of *C. blankii* osteoarticular infection. A. MRI at D140: Osteoarthritis of the metacarpophalangeal joint of the second ray with an abscess in the adjacent soft tissues, measuring approximately 2x2x2 cm, and probable phlegmon around the extensor tendon sheath of the second ray. B. D150 before puncture: swelling of the second metacarpophalangeal joint, suggestive of synovitis and/or arthritis. C. MRI at D232: Extensive intra-articular collection measuring 18 × 13 mm on the palmar side and on the dorsal side measuring 19 × 5 mm (green lines). D. Clinical evolution at D243: Erythematous peri-articular area, seeping opening related to a fistula. (For interpretation of the references to colour in this figure legend, the reader is referred to the Web version of this article.)

On D140, a MRI revealed osteoarthritis of the 2nd metacarpophalangeal left joint with abscesses in the adjacent soft tissues, and a probable phlegmon around the sheath of the extensor tendon (Fig. 1A). On D150, surgical tenosynovectomy was performed at the hand clinic with bone and subcutaneous collection for mycological culture (Fig. 1B). CRP was <6 mg/L and there was no biological inflammatory syndrome. Given the active synovitis, the patient was admitted to the infectious diseases department.

**Fig. 2:**
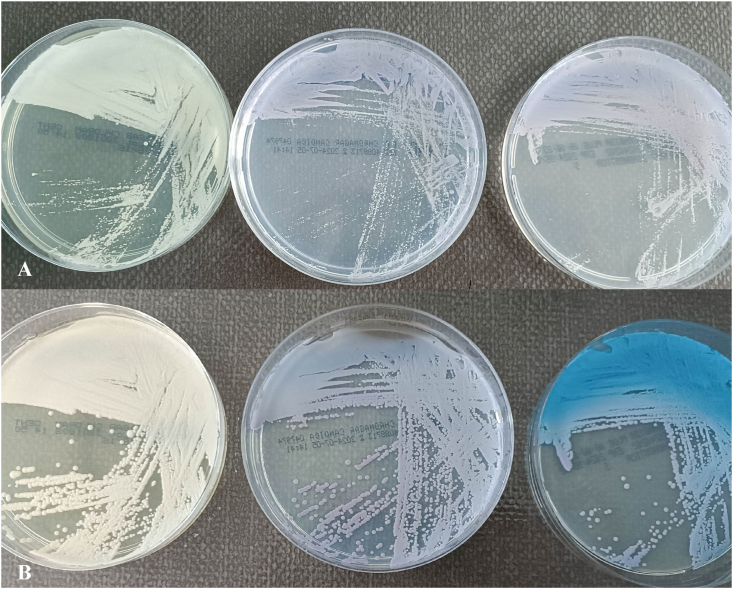
*C. blankii* on (from left to right) Sabouraud, CHROMagar™ Candida and CHROMagar™ Candida Plus at 24h (A) and 48h (B).

The direct examinations of the second puncture were negative. Colonies observed on chromogenic agar (CHROMagar™ Candida) in less than 48 hours, in both bone and subcutaneous collection, were pale pink (Fig. 2). *C. blankii* was identified by MALDI-TOF mass spectrometry (Bruker) with a score of 1.78 and by sequencing of ITS 1/4 regions: 100 % similarity over 474bp with the sequence of the reference strain CBS 6833 (accession KY101965) [6]. Antifungal susceptibility testing using the EUCAST microdilution technique showed MICs of 8 mg/L for fluconazole, 0.5 mg/L for voriconazole, 0.25 mg/L for isavuconazole and 0.5 mg/L for posaconazole. MICs for micafungin and caspofungin were 0.125 mg/L and 0.06 mg/L respectively. MIC was 0.5 mg/L for amphotericin B and ≤0.125 mg/L for 5-FC.

Extension assessment results were negative: negative blood cultures, BD glucan <80 pg/mL, CT-PET with no other deep foci, transthoracic echocardiogram (TTE) and ocular fundus with no signs of infection.

The treatment, initiated one week after surgery (D157), consisted of a combination of voriconazole 200 mg BID (16mg/kg/d) and 5FC 1250 mg QID (104mg/kg/d) for 2 weeks, followed by voriconazole alone for a total of 8 weeks.

After 5 weeks of treatment (D192), a superficial swab of the arthritis was again culture-positive for *C*. *blankii* with similar MICs. Superficial swabs from the left hand after two months of treatment (D217) were negative.

Due to phototoxicity (appearance of forehead skin lesions) and low residual voriconazole levels (0.56 mg/L at D164, 0.69 mg/L at D167, target 1.5–5 mg/L), voriconazole was replaced by posaconazole 300 mg/day after two months' treatment (D222), with the addition of topical voriconazole cream [7].

MRI of the left hand at D232 showed increased metacarpophalangeal osteoarthritis, including adjacent collections, bone erosions of the articular margins with complete decapitation of the metacarpophalangeal joint and complete rupture of the medial band of the extensor tendon at the level of the proximal diaphysis of P1 (Fig. 1C).

Given the poor digestive tolerance of posaconazole and the unfavorable local evolution of the left index arthritis, it was decided to switch the anti-fungal treatment to caspofungin IV (70 mg loading dose on day 1, then 50 mg/d), in combination with 5-FC (100 mg/kg/d PO in 4 doses) from day 228. Corticosteroid therapy at 10mg/d for two days was also added, with progressive tapering (-1mg every 3 days until stopped). Repeated blood cultures were negative. TTE showed no vegetation. B-D-glucan was <80 pg/mL in serum.

At D243 (after 15 days of treatment with caspofungin and 5-FC), the evolution slowly improved with decreased inflammation at the base of the left index finger. A progressive improvement in purulence was observed, as well as closure of the orifice of one of the fistulas (Fig. 1D).

Dual therapy with IV caspofungin and *per os* 5FC was continued at home for a total of 3 months, with a favourable clinical and radiological outcome based on a follow-up at 6 months.

## 3 Discussion

*Candida blankii* was discovered in the 1960s by F. Blank in Canada, in organs of dead minks infected by an unknown yeast. H. R. Buckley and N. van Uden described it in 1968 [8]. It is an ascomycete of the *Saccharomycetaceae* family. It has been found in abundance in coastal marine habitats in Vietnam [9] and in flower nectar in India [10]. *C. blankii* colonies in Sabouraud dextrose agar look like typical yeast, white to cream, with a smooth surface and entire edges. The yeast initially develops pink colonies in CHROMagar Candida Plus and subsequently turned a dark metallic blue, like *C. tropicalis*.

In vitek2 yeast identification system, *C. blankii* was misidentified as *Stephanoascus cifferi* with 89 % probability [4]. Not all MALDITOF MS libraries can identify *C. blankii*. In one study, an in-house MALDI-TOF database was created using the first two molecularly identified strains [1]. In our study, *C. blankii* was identified with a score of 1.78 by the Bruker database MBT Compass reference library (version 2022). However, the best method to identify *C. blankii* seems to be sequence analysis of the internal transcribed spacer 1 and D1/D2 region from the 26S subunit of the rRNA [11].

Recently, a study investigated 26 isolates identified as *C. blankii* by genetical and phenotypical approaches and defined seven strains as a new species: *Tardiomyces depauwii*. This new species is genetically and phenotypically related to *C. blankii*, as it shows a slow growth till 48 h and high MICs to fluconazole and echinocandin [12]. However, *T. depauwii* strains demonstrated elevated voriconazole MICs compared to *C. blankii*. Moreover, because of the large phylogenetic distance of both species to *C. albicans*, the authors proposed the reassignment of *C. blankii* to the genus *Tardiomyces*.

The first case of infection with *C. blankii* was reported between 1990 and 2000 in Norway, during a prospective fungemia study [3]. In 2015, exacerbations due to this yeast in a cystic fibrosis patient were described in Brazil [5]. Fungemia cases have subsequently been reported in immunocompromised patients, including an outbreak in a neonatal intensive care unit in India and in a cystic fibrosis patient, colonized with *C. blankii*, who had just undergone pulmonary transplantation [1,2,4]. This species appears to be quite virulent, being potentially responsible for exacerbations in cystic fibrosis patients, and a possible endocarditis has been described in an immunocompetent patient [13]. Fungemia was also recently reported in a patient infected with SARS-CoV-2 who died [14]. The latest study on *C. blankii*, published in 2023, reports cases of fungemia and otomycosis in Bangladesh [15].

The entry point for *C. blankii* infections is still unknown. Arzumanyan et al. reported cutaneous colonization in a neonate with atopic dermatitis in Russia [16]. In our case, the patient appears to be colonized on skin by *C. blankii*. It could have been inoculated during the infiltration. Although the patient was on isavuconazole prophylaxis, the infection may have developed due to the relatively high MIC of isavuconazole against *C. blankii* (0.25 mg/L) compared to MIC against other *Candida* species such as *C. albicans* (0.008 mg/L). Another explanation could be the poor penetration of isavuconazole at the site of infection. There are currently no data about distribution of isavuconazole in synovial fluid.The patient presented no fever or systemic signs, and the extension workup for fungemia or other deep-seated sites was negative.

Regarding published cases of fungemia in the neonatal intensive care unit, six of the nine neonates had a central venous catheter and four were on mechanically assisted ventilation. *C. blankii* was not found in the vaginal flora of any of the mothers sampled. The origin of the contamination could not be determined [1]. A cutaneous origin is strongly considered, particularly in patients with catheters [14], as *C. blankii* can adhere to plastic surfaces and form biofilms. A respiratory origin may also be considered, as *C. blankii* is capable of colonizing the airways of cystic fibrosis patients.

To our knowledge, there is no report of *C. blankii* infection in France. Interestingly, the present patient reports iterative swimming in a Greek island during summer seasons. This raises the question of whether *C. blankii* is also present nearby European beaches and whether our patient could have colonised her skin in this way.

Yeast infections are less common than those caused by moulds during CGD [7] and bone localizations in particular are predominantly related to *Aspergillus* spp.

In addition, an international meta-analysis published in 2015 did not mention *C. blankii* in joint infections with *Candida* spp [17]. However, 4 % of *Candida* spp strains had not been identified.

The measured MICs are similar to those found in the literature [1,2,12,18]. There are no specific breakpoints for this species. MICs for fluconazole appear to be relatively high (MIC90 = 8 mg/L), while MIC90 for echinocandins are 2, 0.125, and 2 for caspofungin, micafungin, and anidulafungin, respectively.

In published studies, the use of amphotericin B, micafungin, itraconazole and voriconazole has led to favourable outcomes [2,5,13]. Our patient appears to be improving under caspofungin. Echinocandins have been used to treat fungal infections with this species, with a favourable outcome. We can therefore assume that this class of antifungal agent can still be used for deep-seated *C. blankii* infections.

## CRediT authorship contribution statement

**Estelle Sabourin:** Writing – review & editing, Writing – original draft, Investigation, Formal analysis, Conceptualization. **Clémentine De La Porte des Vaux:** Writing – review & editing, Data curation. **Nathanaël Veluppillai:** Formal analysis, Writing – review & editing. **Marie-Elisabeth Bougnoux:** Conceptualization, Data curation, Formal analysis, Writing – original draft, Writing – review & editing. **Eric Dannaoui:** Writing – review & editing, Writing – original draft, Supervision, Methodology, Conceptualization. **Olivier Lortholary:** Conceptualization, Data curation, Formal analysis, Writing – original draft, Writing – review & editing.

## Conflict of interest

There is none.
